# Virosome-masked ratiometric nanoprobes for in vivo dynamic imaging of influenza a virus infection

**DOI:** 10.1186/s12951-026-04819-y

**Published:** 2026-07-21

**Authors:** Ruiqi Ming, Yue Wang, Zewei Yan, Shujun Liu, Zhongjie Wang, Hanlin Chen, Yingjie Shi, Yuantian Jing, Rui Zhang, Shasha Peng, Jiajie Tian, Yimei Pan, Wenfeng Xu, Pengfei Jin, Li-Li Huang

**Affiliations:** 1 School of Medical Science and Engineering, Beijing Institute of Technology, Beijing, 100081 P. R. China; 2 Department of Pharmacy, Beijing Hospital, National Center of Gerontology, Institute of Geriatric Medicine, Beijing Key Laboratory of Assessment of Clinical Drugs Risk and Individual Application, Chinese Academy of Medical Sciences, Beijing, 100730 P. R. China; 3 School of Life Sciences, Inner Mongolia Normal University, Hohhot, 010022 P. R. China

**Keywords:** Virosome, Ratiometric nanoprobe, Signal amplification, Spatiotemporal tracking, In situ monitoring

## Abstract

In vivo fluorescent in situ visualization of viral infection dynamics is crucial for elucidating the mechanisms of viral pathogenesis. However, current approaches lack sufficient specificity and sensitivity for spatiotemporal monitoring of viral infection in vivo. Here, we proposed virosome-masked ratiometric nanoprobes (VMR-NPs) integrated with dual-stimuli-responsive near-infrared (NIR) Förster resonance energy transfer (FRET) reporters that specifically recognize influenza A virus (IAV) viral RNA (vRNA) and apurinic/apyrimidinic endonuclease 1 (APE1). Surface hemagglutinin (HA) envelope protein of IAV virosome endows VMR-NPs with enhanced host-cell association and IAV-like intracellular trafficking behavior. Following internalization, vRNA recognition together with APE1-assisted signal amplification modulates the ratiometric fluorescence output of VMR-NPs, enabling sensitive detection of infection-associated signal changes in vitro and in vivo. This study establishes an IAV virosome-masked sensing platform for imaging infection-associated IAV burden and provides a useful tool for studying virus-related biological processes.

**Graphical Abstract:**

**Figure:**
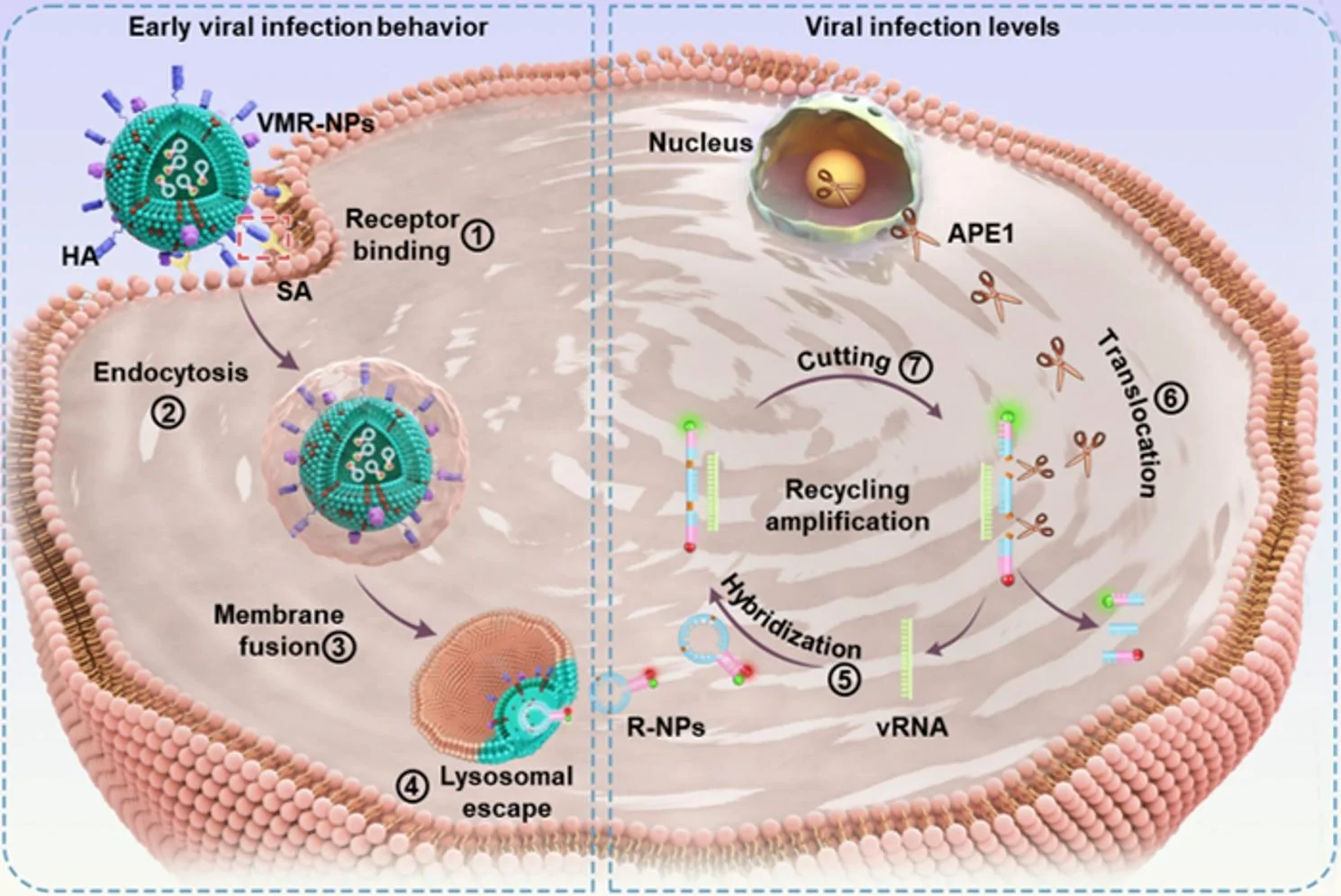

**Supplementary Information:**

The online version contains supplementary material available at https://doi.org/10.1186/s12951-026-04819-y.

## Introduction

The emergence of highly infectious and pathogenic viruses, such as novel coronaviruses and influenza viruses (IAVs), poses a significant threat to public health, socio-economic stability, and national security [1]. Comprehensive and in-depth understanding of the mechanisms of viral infection is imperative for unraveling pathogenicity, and guiding the development of diagnostic tools and therapeutic interventions [2]. However, viral infection is a complex cascade of spatiotemporally dynamic events [3, 4], creating an urgent demand for advanced in vivo imaging technologies and nanoprobes that enable real-time, in situ tracking of these processes while preserving native virus-host interactions.

Current in vivo fluorescent imaging strategies for viral infection suffer from three critical limitations that impede their translational potential [5, 6]. First, direct conjugation of fluorophores to viral surfaces often obstructs key recognition sites, potentially altering viral infectivity and/or canonical infection pathways [7, 8]. Second, most in vivo single-signal fluorescent probes are inherently susceptible to external environmental factors, leading to signal fluctuations and inaccurate detection [9, 10]. Third, the biological complexity of living organisms hampers high-resolution spatiotemporal tracking of viral infection and precise quantification of viral load and replication at viral infection lesions [11, 12]. Thus, there is a need for imaging probes that enable sensitive and time-resolved visualization of infection-associated molecular events in vivo while minimizing interference with probe performance and preserving biologically relevant host–pathogen interactions as much as possible.

**Scheme 1:**
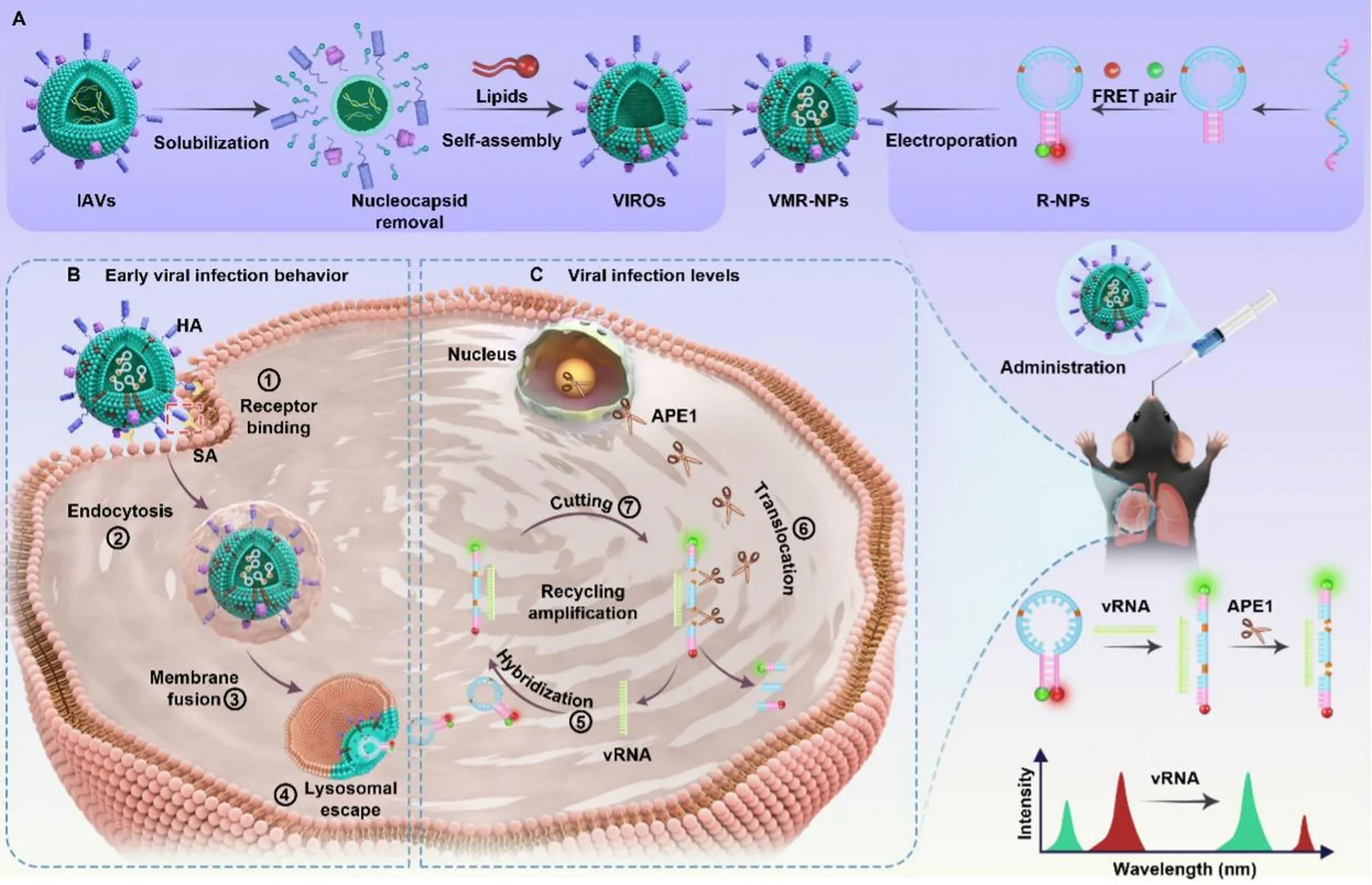
Schematic illustration of the preparation (**a**) and in vivo monitoring of viral infection (**b-c**) of VMR-NPs

To address this challenge, we developed virosome-masked ratiometric nanoprobes (VMR-NPs) that combine HA-mediated host-cell recognition with dual-triggered fluorescence readout for imaging infection-associated IAV signals in vivo. VMR-NPs adopt a “sensing core-viral mimic shell” structure where ratiometric nanoprobes (R-NPs) serve as the core while influenza virosomes (VIROs) retaining native viral envelope proteins form the shell. The R-NPs are composed of two NIR fluorophores with Förster resonance energy transfer (FRET) activity connected by a molecular beacon (MB) that can simultaneously respond to viral RNA (vRNA) of IAVs and apurinic/apyr-imidinic (AP) endonuclease 1 (APE1) (Scheme 1a). The surface hemagglutinin (HA) envelope proteins endow VMR-NPs with high-affinity for host cells and effective endosomal escape, which can not only mimic the HA-dependent early IAV infection behavior (including viral attachment, endocytosis and endosomal escape) via ratiometric NIR fluorescence (FL) imaging (Scheme 1b), but also boost R-NPs release into the cytosolic compartment of IAV-infected host cells. Subsequently, vRNA binding to the MB and site-specific cleavage by APE1 synergistically trigger activation and amplification of the R-NP donor fluorophore signal, with a concurrent decrease in the acceptor signal. This ratiometric readout enables sensitive and specific in situ monitoring of viral load via FL imaging in IAV-infected host cells (Scheme 1c). Overall, this approach offers a novel platform for specific, sensitive monitoring of key molecular events in IAV infection and pathogenicity based on HA-mediated IAV-like targeting and dual-signal sensing.

## Experimental section

### Materials

Roswell Park Memorial Institute 1640 (RPMI 1640), phosphate-buffered saline (PBS), fetal bovine serum (FBS) and 0.25% trypsin-ethylenediaminetetraacetic acid (EDTA) were obtained from Gibco (NY, USA). Penicillin-streptomycin was acquired from Macgene (Beijing, China). All DNA sequences were synthesized and HPLC purified by Sangon Biotech Co. Ltd. (Shanghai, China). PrimeScript™ Ⅱ 1st Strand cDNA Synthesis Kit were purchased from TransGen Biotech (beijing, China). Octaethyleneglycol-mono-(n-dodecyl) ether, leptomycin B and apurinic/apyrimidinic endonuclease1 (APE1) were purchased from MedChemExpress (Shanghai, China). Anti-H9N2 HA protein antibody was purchased from Thermo (MA, US). Bicinchoninic acid (BCA) protein assay kit, electrogenerated chemiluminescence (ECL), Cy5, Cy7-NHS, ActivAb^TM^Goat Anti-Rabbit IgG/HRP and Lyso-Tracker Green were purchased from Solarbio Science & Technology Co. Ltd. (Beijing, China).

### Cell culture

A549 cells were cultured in Dulbecco’s modified Eagle’s Medium (DMEM, Gibco) with 10% FBS, penicillin (100 mg/mL) and streptomycin (100 mg/mL) at 37 °C in an environment with 5% CO_2_.

### R-NPs and target vRNA sequences

BLAST (https://blast.ncbi.nlm.nih.gov/Blast.cgi) was used to select the target sequences that are unique for the target vRNA. R-NPs sequence: 5′-/Cy7/ C*G*C*A*C*C*C*A*TGAATTCA/idSp/GTGAGGTT/idSp/GAAACTAG*A*C*G*G*T*G*C*G/Cy5/-3′. The non-targeting control nR-NPs sequence: 5′-/Cy7/C*G*C*A*C*C*C*A*GAAATATG/idSp/TTGTTGCC/idSp/GAATCTGA*A*C*G*G*T*G*C*G/Cy5/-3′. The bold letters represent the bases in the stems of the beacon; ‘idsp’ indicates the AP sites on the loop; ‘*’ denotes phosphorothioate bonds; the underlined region identifies the targeting sequence. The beacon is labeled with Cy7 at the 5′ end and Cy5 at the 3′ end, respectively. Target vRNA sequence: 5′-GUCUAGUUUCAACCUCACUG AAUUCAUG-3′.

### APE1-triggered amplified vRNA sensing in vitro

The R-NPs were prepared in an annealing buffer (pH 7.4, 20 mM HEPES, 150 mM NaCl, 5 mM MgCl_2_). The solution was incubated at 95 °C for 5 min and then slowly cooled down to room temperature and stored at 4 °C for further use. The vRNA sensing assays were performed in 100 µL of reaction buffer (pH 7.4, 50 mM HEPES, 50 mM NaCl, 10 mM MgCl_2_ and 0.1 mg/mL BSA). For monitoring of molecular beacon opening, 1 µM R-NPs were incubated with 2 µM vRNA of IAV H9N2 strain in the absence of APE1 (Cat. No. HY-P7523) and then subjected for fluorescence monitoring. For fluorescence comparison assay, 0.5 µM R-NPs were incubated with 0.1 µM of vRNA and 0.8 µg/mL of APE1 at 37 °C for 40 min. The emission spectra ranging from 530 nm to 750 nm were collected by excitation at 520 nm.

### Virus propagation and virus titer assays

The influenza A virus H9N2 strains were obtained from Wuhan Institute of Virology, Chinese Academy of Sciences, and propagated in the allantoic cavity of 10 days old embryonated eggs for 48 h at 37 °C. Subsequently, viruses were purified as previously described [13].

The titer of viruses was quantified by TCID_50_. Specifically, MDCK cells were cultured in 96 well plates in culture medium until the cells reached 80 − 90%. The virus samples and their paired controls were immediately serially diluted with DMEM supplemented with 0.1% FBS, 0.3% bovine serum albumin (BSA, Gibco, USA) and 1% antibiotics. They were subsequently added to the cells and infected for 1 h. TCID_50_ cultures were washed and fed with DMEM containing 2% FBS. Then the infected cells were cultured in an incubator at 37 °C with CO_2_ for about 5 days. The number of wells that had cytopathic effect (CPE) on MDCK cells was counted. TCID_50_ was calculated from the Reed‒Muench formula: TCID_50_ = dilution above 50% CPE + [(% next above 50%) − 50%] / (% next above 50% − (% next below 50%) × log10.

### Preparation of VIROs

4 mg of egg phosphatidylcholine (PC) and 1 mg of 1-oleoyl-3-palmitoyl-rac-glycero-2-phosphoethanolamine (PE) were dissolved in 400 µL of PBS containing 100 mM octaethyleneglycol-mono-(n-dodecyl) ether (OEG-PBS). 2 mg of HA-contained IAVs were dissolved in 1 mL of OEG-PBS. The detergent-solubilized phospholipids and IAVs were mixed and sonicated for 1 min. This mixture was centrifuged at 110,000 × g for 2 h at 4 °C. The supernatant was used, and VIROs were formed by detergent removal using 625 mg of wet SM2 Bio-Beads (BioRad) for 1 h at room temperature, followed by three additional rounds of treatment with 300 mg of SM2 Bio-Beads each for 30 min. Finally, the VIROs were sterile filtered through 0.22 mm filter.

### Preparation of VMR-NPs

Different doses of R-NPs (0.25 µM, 0.5 µM, 1 µM, 2 µM and 4 µM) were mixed with 10 µg VIROs in 200 mL PBS, and transferred to a 4 mm electroshock cup. Electrotransformation was performed at 100 V, 100 µF and 200 Ω. Then, the sample was incubated on ice for 30 min and centrifuged at 110,000 × g for 2 h at 4 °C to remove free R-NPs. After purification, the fluorescence intensity of VMR-NPs was measured using a fluorescence spectrophotometer and compared with a standard curve generated from known concentrations of free R-NPs. The concentration of VMR-NPs was defined according to the amount of R-NPs loaded inside VIROs. The hydrodynamic diameter distribution of VMR-NPs was determined by DLS (Malvern, UK). The morphology of VMR-NPs was characterized by TEM (HITACHI, Japan).

Loading Efficiency = (weight of loaded R-NPs) / (weight of total R-NPs and VIROs) × 100%.

Encapsulation Efficiency = (weight of loaded R-NPs) / (weight of total R-NPs) × 100%.

### SDS‒PAGE and western blotting analysis

For SDS-PAGE protein profiling, IAVs, VIROs, and VMR-NPs were mixed with loading buffer and heated at 100 °C for 10 min. Equal amounts of total protein were loaded onto 10% polyacrylamide gels and separated by electrophoresis. The gels were then subjected to total protein staining to visualize the major viral protein bands. The HA, NA, NP, and M bands were assigned according to their apparent molecular weights and comparison with the protein profile of purified IAVs. In addition, separate western blotting experiments were performed using specific anti-HA and anti-NA antibodies to further confirm the retention of HA and NA proteins in VIROs and VMR-NPs. Briefly, after SDS-PAGE, proteins were transferred onto PVDF membranes. The membranes were blocked with 5% BSA, incubated with primary antibodies overnight at 4 °C, and then incubated with HRP-conjugated secondary antibodies. Protein signals were visualized using ECL reagent.

### Colocalization of VMR-NPs

The R-NPs were loaded into VIROs. Then, VIROs were labeled with 1,1′-dioctadecyl-3,3,3′,3′-tetramethylindocarbocyanine perchlorate (DiO) for 30 min at room temperature. The free fluorescent molecules were removed by ultrafiltration. Then, DiO-labeled VMR-NPs were dropped on a glass slide, covered with a coverslip, and visualized using laser scanning confocal microscope (CLSM, Nikon, Japan). DiO was excited at 488 nm, and R-NPs were excited at 633 nm. The tMr (tMg) value indicate the percentage of the red (green) signals colocalized with green (red) signals in the corresponding thresholded image, ICQ quantifies the degree of pixel-by-pixel intensity correlation between two channels, with values ranging from − 0.5 (negative correlation) to + 0.5 (positive correlation).

### Real‒time the individual IAV or VMR-NPs in a cell

For live-cell trajectory tracking, VMR-NPs and LR-NPs were monitored through the Cy5 fluorescence signal of the encapsulated Cy3/Cy5 ratiometric probe. Native IAV particles were labeled with DiI and tracked in the DiI channel. A549 cells were incubated with DiI-labeled IAVs, VMR-NPs, or LR-NPs. Time-lapse images were acquired at 500 ms intervals for approximately 36 min using a live-cell imaging chamber maintained under physiological culture conditions. Trajectory analysis of live-cell imaging included only continuously traceable particles and excluded cells with obvious displacement or deformation. For trajectory-resolved analysis, single-particle tracks were segmented into five consecutive stages according to characteristic changes in instantaneous velocity profiles. Each segment was then analyzed by fitting the corresponding MSD-versus-time curve to distinguish confined/anomalous diffusion from directed transport. Directed velocity (V) and diffusion coefficient (D) were calculated from the fitted models for the relevant stages. For trajectory analysis, approximately 10–14 particle trajectories from 5 to 7 cells were analyzed per group. MSD was calculated by the user-written program with MATLAB software. The perinuclear FIP was calculated as the integrated FL intensity within the perinuclear region divided by the total FL intensity of the whole cell × 100%.

### Endosomal escape of VMR-NPs

A549 cells (1 × 10^5^ cells/mL) were seeded in confocal dishes (Biosharp, Anhui, China) and cultured overnight at 37 °C. Then, the cells were treated with VMR-NPs for 1, 2, 4, and 6 h, respectively. Subsequently, the cells were washed three times with PBS, and the lysosomes were labeled with 50 nM Lyso-Tracker green. The cell nucleus was labeled with Hoechst 33,342. Finally, the cells were washed with PBS, and fluorescence images were taken by CLSM. Co-localization between VMR-NPs and lysosomes was quantified using Pearson’s correlation coefficient. Fluorescence thresholds for each channel were determined automatically using the Costes thresholding algorithm [14]. At least 10 cells were analyzed for each group/time point. Representative scatter plots and fluorescence intensity profiles were generated from individual cells to visualize the pixel-wise correlation between the two channels.

### In vitro ratiometric FL imaging of IAV infection

For intracellular IAV infection imaging, A549 cells (1 × 10^5^ cells/well) were seeded in confocal dishes and incubated at 37 °C with 5% CO₂ for 12 h. After cell adhesion, the original medium was aspirated and replaced with fresh medium containing IAVs (MOI = 0, 10^− 3^, and 10) and VMR-NPs (final concentration of 1 µM). The cells were further incubated for 2, 4, 6, and 8 h, respectively. At each time point, ratiometric fluorescence images were acquired using a fluorescence microscope with excitation at 550 nm. Cy3 emission was collected within 560–600 nm, and Cy5 emission was collected within 660–700 nm.

In addition, for virus proliferation monitoring, A549 cells (2 × 10^4^ cells/well) were seeded in confocal dishes and incubated in a 37 °C, 5% CO₂ incubator for 12 h. The lower initial cell density was used to maintain appropriate cell density and cell status during the 72 h observation period. After the cells adhered to the plate, the original medium was replaced with fresh medium containing IAVs (MOI = 10⁻³) and VMR-NPs (final concentration of 1 µM). The cells were then further incubated for 2, 6, 12, 24, 48, and 72 h, respectively. At each of the aforementioned time points, ratiometric fluorescence images were acquired using the same excitation/emission settings described above.

### Animal experiments and ethical permits

All animal experiments were performed under the guidance of the Ethics Committee of Beijing Institute of Technology (approval ID: 2019-0010-M-036). C57BL/6 mice (male, 6 weeks) were purchased from Vital River Laboratory Animal Technology Co., Ltd (Beijing, China).

### Imaging of IAVs and VMR-NPs metabolism

IAVs were labeled with Cy7-NHS (N-hydroxysuccinimide ester) via amide bond formation. Both VMR-NPs and Cy7-labeled IAVs at the same concentration were intratracheally administered into mice. Images were captured at 0, 2, 8, 12, 24, 48 and 72 hpi by in vivo animal imaging system (PerkinElmer, USA) (excitation wavelength: 650 nm; emission wavelength: 750 nm). The major organs were dissected and subjected for ex vivo imaging. Average radiant efficiency of VMR-NPs and Cy7-labeled IAVs was quantified by Living Image analysis software.

### RT-qPCR analysis of viral RNA

Lung tissues were collected and homogenized for total RNA extraction. After reverse transcription, quantitative PCR was performed to detect IAV viral RNA. The Ct values of viral RNA were normalized to an internal reference gene, and relative viral RNA levels were calculated using the 2^-ΔΔCt method. The virus-free group was used as the calibrator control for relative quantification.

### In vivo ratiometric NIR FL imaging of IAV infection

For dose-dependent in vivo imaging, mice were intratracheally administered 100 µL of a mixture containing IAV at different inoculum doses (0, 10^2^, or 10^6^ PFU/mouse) together with VMR-NPs (final concentration, 10 µM). Images were captured at 0 and 16 hpi using an in vivo animal imaging system (PerkinElmer, USA) with excitation at 650 nm. Cy5 emission was collected within 660–700 nm, and Cy7 emission was collected within 760–820 nm. After in vivo imaging, lungs were dissected and subjected to ex vivo imaging. The average radiant efficiency of VMR-NPs was quantified using in vivo imaging system (IVIS).

In addition, for serial in vivo imaging under low-dose infection conditions, mice were first intratracheally administered 100 µL of IAV suspension at 10^2^ PFU/mouse. VMR-NPs (final concentration of 10 µM) were administered to mice via intratracheal injection 16 h prior to each imaging time point. Images were captured at 16, 28, 52, and 100 hpi using the same Cy5/Cy7 excitation/emission settings and quantitative procedures described above. After in vivo imaging, lungs were dissected and subjected to ex vivo fluorescence imaging. The average radiant efficiency of VMR-NPs in lungs was quantified using IVIS.

### Statistical analysis

All data are presented as the mean ± standard deviation (SD), unless otherwise indicated. Statistical analyses were performed using GraphPad Prism 8. Unpaired two-tailed Student’s t test or one-way analysis of variance (ANOVA) with Sidak’s multiple comparisons test was used as appropriate. n.s.: not significant, *P < 0.05, **P < 0.01, ***P < 0.001, and ****P < 0.0001.

## Results and discussion

### Construction and characterization of VMR-NPs

**Fig. 1:**
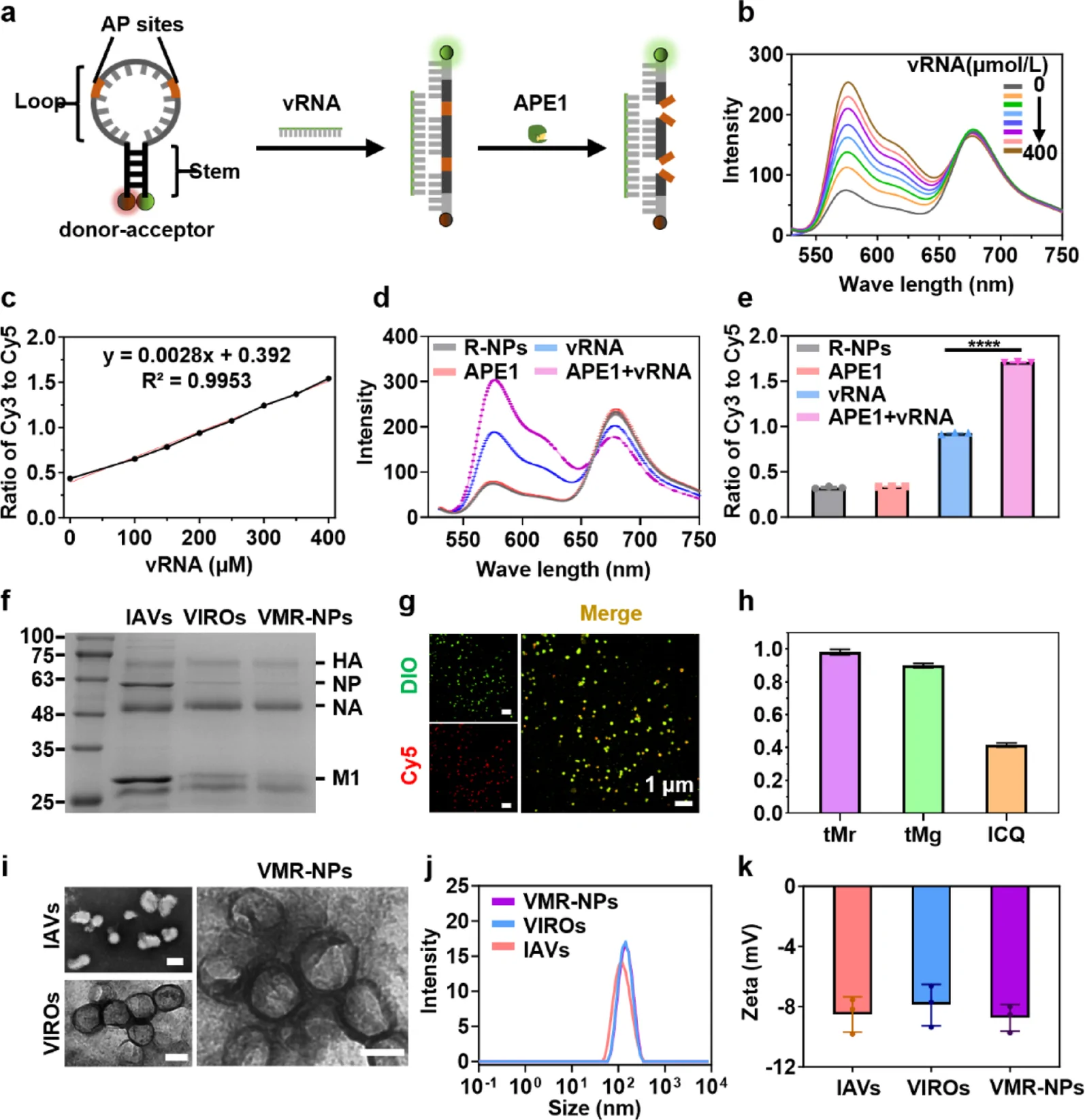
Construction and characterization of VMR-NPs. **a**) Schematic illustration of the design and working principle of the R-NPs. **b**) Fluorescence emission spectra of R-NPs in response to different concentrations of target vRNA in the absence of APE1, with R-NPs without target vRNA included as the baseline control. **c**) Linear relationship of the FL_ratio_ and vRNA concentration. **d-e**) FL spectra and quantitative analysis of the FL_ratio_ of R-NPs in response to vRNA alone or the combination of vRNA and APE1. **f**) SDS-PAGE profiling of protein components in VMR-NPs, VIROs and IAVs. **g-h**) Confocal FL imaging (**g**) and co-localization analysis (**h**) of R-NPs (red) and DiO-labeled VIROs (green). Scale bar: 1 μm. **i**) TEM images of IAVs, VIROs and VMR-NPs. Scale bar: 100 nm. **j-k**) Dynamic light scattering (DLS) analysis of hydrodynamic size distribution (**j**) and zeta potential measurements (**k**) of VMR-NPs. Data are presented as mean ± standard deviation (*n* = 3). Statistically significant differences between groups were identified by one-way ANOVA with Sidak’s multiple comparisons test. ****P < 0.0001

To establish a vRNA-responsive detection system, we engineered R-NPs integrated with a MB functionalized with a Cy3-Cy5 FRET donor-acceptor pair for intracellular applications. This MB possesses a canonical stem-loop architecture, a 6-bp stem, a 16-nt loop containing two abasic (AP) sites, and Cy3/Cy5 conjugated to its opposing termini. The closed conformation of the stem region brings Cy3 and Cy5 into close proximity, enabling efficient FRET. Upon hybridization with complementary vRNA, the stem-loop structure undergoes a conformational switch to the open state, abrogating FRET and consequently restoring the donor fluorophore emission while quenching the acceptor signal (Figure S1, Supporting Information). Notably, APE1 can specifically cleave the AP sites on the MB, which triggers the release of target vRNA. The freed vRNA then binds to other intact MBs, initiating a cyclic hybridization-cleavage reaction to form a target-recycling amplification cascade (Fig. 1a) [15, 16]. The Cy3/Cy5 FL intensity ratio (FL_ratio_) showed a linear correlation with increasing target vRNA concentrations (Fig. 1b, c), confirming the R-NPs enable dynamic, quantitative vRNA detection. A non-targeting molecular beacon control (nR-NPs) showed no obvious spectral change or FL_ratio_ increase upon incubation with the same target vRNA, confirming that R-NP activation depends on sequence-specific vRNA recognition (Figure S2, Supporting Information). It is worth emphasizing that APE1 is highly expressed in virus-infected cells and translocates from the nucleus to the cytoplasm [15, 16]. This APE1-mediated signal amplification strategy remarkably boosts the donor fluorescence intensity, with the FL_ratio_ increased by 1.87-fold compared to the scenario where only vRNA is present (Fig. 1d, e). Furthermore, this amplification effect was concentration-dependent (Figure S3, Supporting Information), validating that APE1 boosts vRNA detection sensitivity via signal amplification. Linear calibration curves for low vRNA concentrations (0–50 nM) were used to determine the limit of detection (LOD), which was calculated to be 2.06 nM (Figure S4, Supporting Information).

To further develop a functional IAV virosome-masked ratiometric nanoprobes, we fabricated recombinant viral envelope vesicles (VIROs) from IAV membranes while removing viral nucleic acids and nucleocapsid-associated components (Figure S5, Supporting Information) [17, 18]. SDS-PAGE showed that four major bands were observed, among which hemagglutinin (HA), and neuraminidase (NA), and matrix (M) protein were clearly identifiable, whereas the nucleoprotein (NP) band was largely absent in the VIROs/VMR-NPs samples (Fig. 1f). Western blot analysis further confirmed the presence of HA and NA in VIROs (Figure S6, Supporting Information). The R-NPs were loaded into VIROs via electroporation to generate VMR-NPs, followed by ultracentrifugation to remove free R-NPs. Spectral analysis confirmed that the loading efficiency was approximately 39% (Figure S7, Supporting Information). Co-localization imaging further supported successful loading of R-NPs into DiO-labeled VIROs, with high co-localization efficiency (tMr: 0.97 ± 0.05, tMg: 0.91 ± 0.07, intensity correlation quotient (ICQ): 0.45 ± 0.04) (Fig. 1j, k). Collectively, these data confirm the successful integration of sensing components into the IAV virosomes. VMR-NPs exhibited a typical spherical morphology, consistent with both native IAVs and VIROs (Fig. 1g). Their hydrodynamic diameter and zeta potential averaged 120 ± 13.0 nm and − 10 ± 0.7 mV, respectively (Fig. 1h, i), and remained stable in 10% fetal bovine serum (FBS) over 7 days (Figure S8 and S9, Supporting Information).

### IAV-like host-cell association and intracellular trafficking of VMR-NPs

**Fig. 2:**
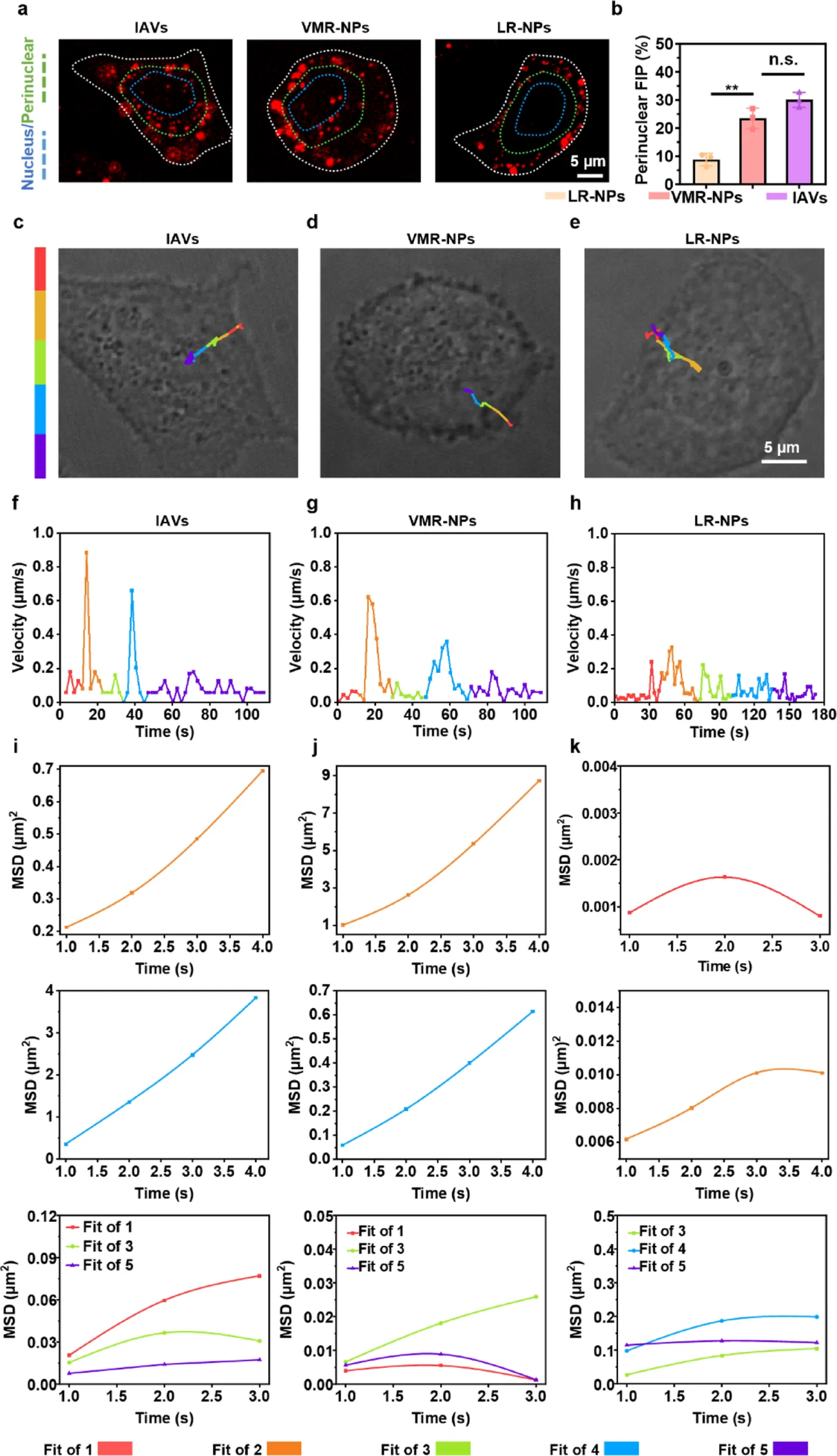
Time-lapse tracking of intracellular trafficking behaviors of labeled IAV particles, VMR-NPs, and LR-NPs in A549 cells. **a**) FL imaging of A549 cells treated with DiI-labeled IAVs, VMR-NPs, or LR-NPs for 36 min. Dashed lines represent the whole-cell boundary (white), the perinuclear region (green), and the nuclear region (blue). Scale bar, 5 μm. **b**) Quantification of perinuclear fluorescence intensity percentage (FIP). **c-e**) Trajectory of an IAVs, VMR-NPs and LR-NPs inside host cells. Scale bar: 5 μm. **f-h**) Instantaneous velocity profiles of the representative trajectories shown in (c-e), used for stage segmentation. **i-k**) Stage-wise MSD–time plots of IAVs (i), VMR-NPs (j) and LR-NPs (k). Directed velocity (V) and diffusion coefficient (D) were calculated for the indicated stages. Data are presented as mean ± standard deviation (*n* = 3). Statistically significant differences between groups were identified by one-way ANOVA with Sidak’s multiple comparisons test. n.s. not significant, **P < 0.01

VMR-NPs possess certain nanoscale physicochemical characteristics of IAVs and retain HA on their surface, leading us to hypothesize that they may exhibit enhanced host-cell association and IAV-like internalization behavior [19–21]. To examine this hypothesis, we performed real-time imaging to compare the entry dynamics of VMR-NPs and native IAVs in human lung epithelial (A549) cells, with liposome-loaded R-NPs (LR-NPs) as a negative control. As expected, VMR-NPs exhibited IAV virosome-mediated entry kinetics highly similar to native IAVs. Both rapidly attached to the cell surface and then gradually internalized into perinuclear region over a 36-minute period via the same IAV virosome-dependent pathway. In comparison, LR-NPs, which lack HA-mediated receptor recognition, their inward transport was less coordinated and their perinuclear accumulation was weaker than those of IAVs and VMR-NPs under the same imaging conditions (Fig. 2a, b, Figure S10, Supporting Information). This highlights the critical role of IAV virosome in mediating IAV–host cell interactions and underscores the IAV virosome-mediated functional mimicry of VMR-NPs in simulating the early entry process of authentic IAVs (attachment and endocytosis).

To further characterize the intracellular transport behavior of these particles, we reconstructed single-particle trajectories of IAVs, VMR-NPs, and LR-NPs via time-lapse imaging data. As shown in Fig. 2c, d and Movies S1, S2, both VMR-NPs and labeled IAVs exhibited coordinated inward movement from the cell periphery toward the perinuclear region over time. This transport pattern is consistent with HA-mediated receptor-associated endocytic uptake and subsequent endosomal trafficking toward the perinuclear region. In contrast, LR-NPs displayed more irregular and less directional trajectories (Fig. 2e, Movie S3). Although nonspecific cellular uptake or nanoparticle-associated endocytosis may still occur, the absence of HA-mediated receptor recognition likely reduced the engagement of LR-NPs in IAV-like endosomal trafficking. As a result, LR-NPs mainly showed diffusion-dominated or confined motion, with limited long-range inward transport. For stage-resolved analysis, trajectories were segmented according to changes in instantaneous velocity profiles, and the corresponding mean-squared displacement (MSD)–time curves were fitted to classify the dominant motional features in each segment (Fig. 2f–k). On this basis, both labeled IAV particles and VMR-NPs could be described by a similar five-stage intracellular transport pattern [13, 22, 23]. Stage 1 corresponded to slow peripheral confined motion after cell surface binding/internalization, with instantaneous velocities below ~ 0.2 μm·s⁻¹ and MSD-τ curves consistent with anomalous diffusion (VMR-NPs, α < 1, D₁≈0.0013 μm²·s⁻¹; IAVs, α < 1, D₁≈0.0089 μm²·s⁻¹). Stage 2 represented the first inward directed transport phase, in which the MSD-τ curve exhibited a marked upward curvature and were better fitted by a directed-motion model (VMR-NPs, V₂ ≈ 0.344 μm·s⁻¹, D₂ ≈ 0.102 μm²·s⁻¹; IAVs, V₂ ≈ 0.074 μm·s⁻¹, D₂ ≈ 0.0035 μm²·s⁻¹). Stage 3 was characterized by slower bidirectional oscillatory motion in the cytoplasm, with plateauing MSD-τ curves consistent with confined or anomalous diffusion (VMR-NPs, D₃ ≈ 0.0056 μm²·s⁻¹, α < 1; IAVs, D₃ ≈ 0.0105 μm²·s⁻¹, α < 1). Stage 4 corresponded to a second inward directed transport phase toward the deeper perinuclear region (VMR-NPs, V₄ ≈ 0.058 μm·s⁻¹, D₄ ≈ 0.0117 μm²·s⁻¹; IAVs, V₄ ≈ 0.135 μm·s⁻¹, D₄ ≈ 0.081 μm²·s⁻¹). In stage 5, both particles became relatively confined in perinuclear region, with net displacements < 0.5 μm over several minutes and the MSD-τ curve was again consistent with anomalous diffusion (VMR-NPs, D₅ ≈ 0.0058 μm²·s⁻¹, α ≈ 0.75; IAVs, D₅ ≈ 0.0228 μm²·s⁻¹, α ≈ 0.6). In contrast, LR-NPs exhibited a clearly different motional pattern. Their instantaneous velocities remained low with only slight irregular fluctuations, and their MSD-τ curves lacked the upward-bending segments associated with directed transport in IAVs/VMR-NPs. Instead, LR-NPs were better described by low-mobility diffusion-dominated behavior (D ≈ 0.00021 μm²·s⁻¹, α < 1), with no clear evidence of long-range inward transport or stable perinuclear confinement (Fig. 2h, k). These quantitative data collectively confirm that VMR-NPs mimic the spatiotemporal dynamics of native IAVs in the IAV virosome-dependent early processes, from initial host cell recognition and entry to early intracellular transport toward the perinuclear region. In conclusion, VMR-NPs provide an IAV virosome-assisted sensing platform for investigating selected early infection-associated cellular events with reduced biosafety concerns.

**Fig. 3:**
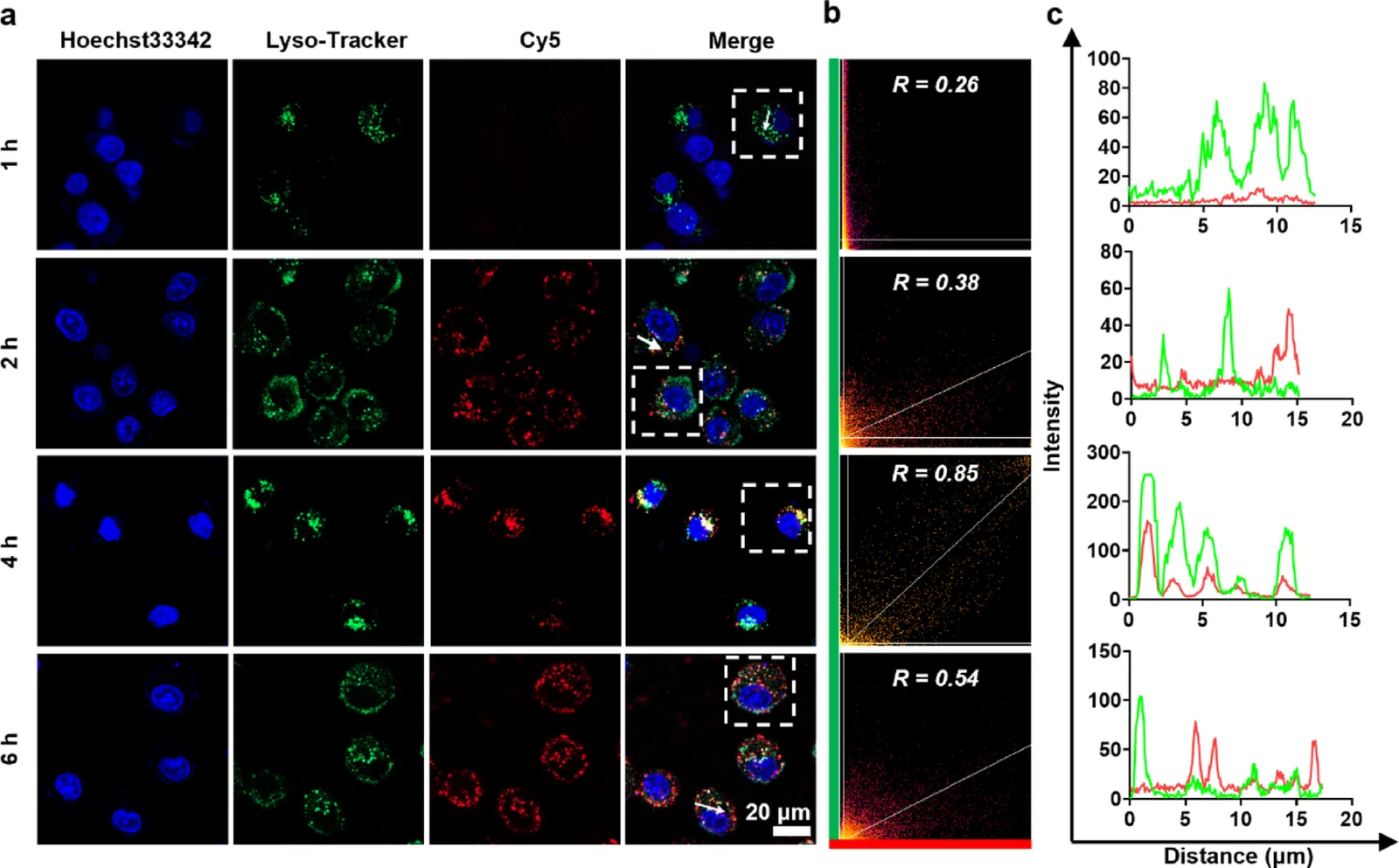
Time-dependent lysosomal escape of VMR-NPs in A549 cells. **a**) Representative confocal fluorescence imaging of A549 cells treated with VMR-NPs for the indicated time, followed by staining with Lyso-Tracker (green) and Hoechst 33342 (blue). **b**) Representative whole-cell scatter plot analysis used for co-localization quantification. R value was calculated after automatic thresholding using the Costes algorithm. **c**) Fluorescence intensity profiles of green and red channels along a selected line across representative cells

After IAV internalization, the HA protein mediates the fusion of the viral envelope with the endosomal membrane, promoting the release of the viral nucleocapsid into the cytoplasm for replication [24, 25]. Given the abundant HA displayed on the surface of VMR-NPs, we next examined their lysosomal escape behavior after cellular uptake. As shown in Fig. 3a, VMR-NPs exhibited substantial co-localization with LysoTracker at 4 post-incubation (hpi), whereas the overlap was markedly reduced by 6 hpi, indicating progressive escape from lysosomes over time. To quantify this change, we performed whole-cell co-localization analysis using Pearson’s correlation coefficient (R), with thresholds for each channel determined by the Costes automatic thresholding algorithm. The decrease in R values at later time points further supported the time-dependent lysosomal escape of VMR-NPs and their increasing redistribution into the cytoplasm (Fig. 3b, c).

### Ratiometric FL imaging of IAV infection in host cells

**Fig. 4:**
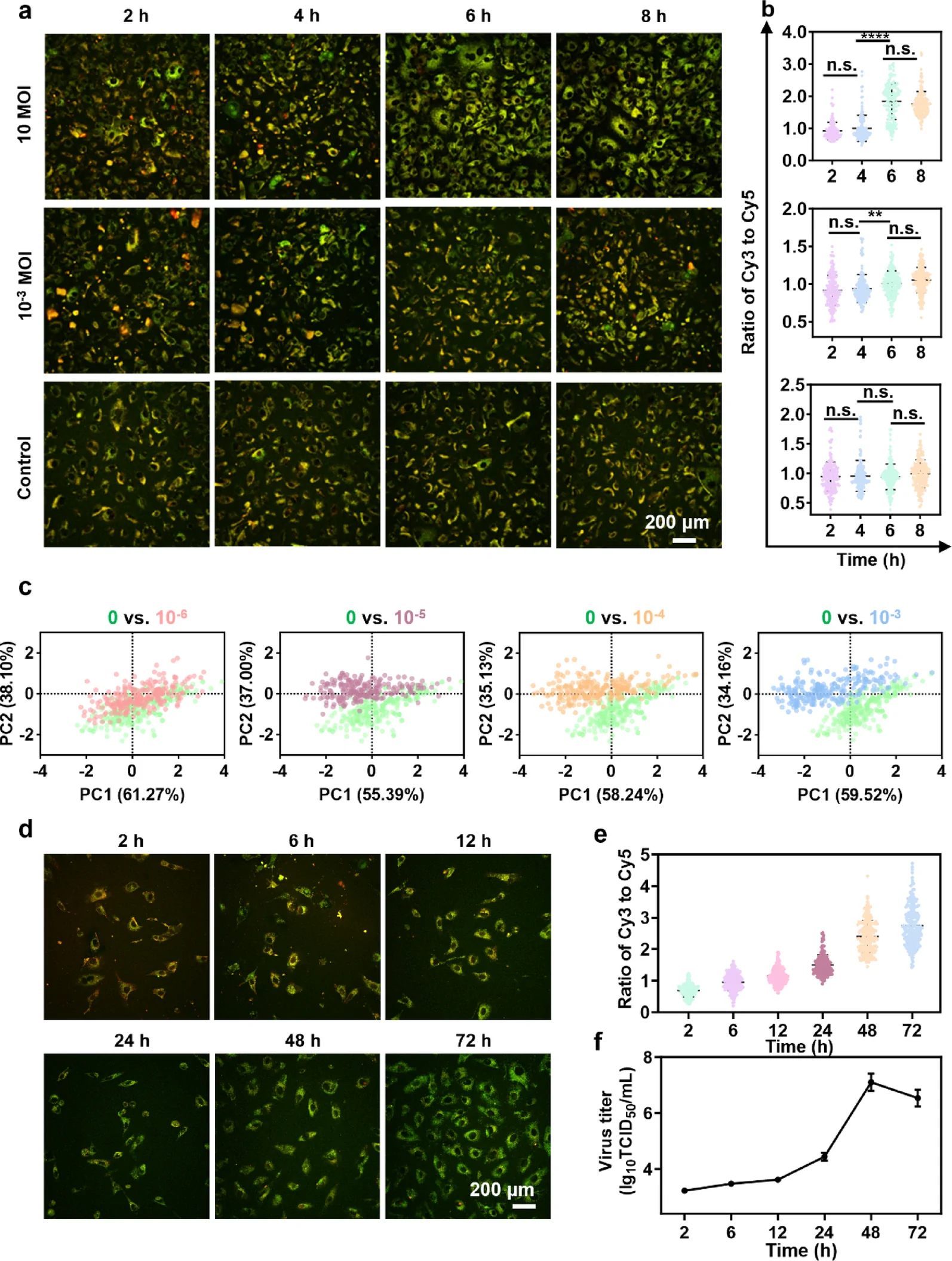
In vitro ratiometric imaging of infection-associated IAV signals using VMR-NPs. **a-b**) Confocal FL images (**a**) and quantitative FL_ratio_ analysis (**b**) of VMR-NPs in A549 cells infected with IAVs at distinct viral titers and incubation time points. **c**) PCA score plots showing separable clustering among uninfected cells (MOI = 0) and cells infected with different IAV titers (MOI = 10⁻⁶ to 10⁻³); variance contributions of principal component 1 (PC1) and PC2 are indicated. **d-e**) FL images (**d**) and quantitative FL_ratio_ analysis (**e**) of VMR-NPs monitoring IAV-infected cells at different time points. **f**) Time-dependent change in viral titer determined by TCID₅₀ assay in matched infected-cell samples (MOI = 10⁻³). Data are presented as mean ± standard deviation (*n* = 3). Statistically significant differences between groups were identified by one-way ANOVA with Sidak’s multiple comparisons test. n.s. not significant, **P < 0.01, ****P < 0.0001

We first confirmed that VMR‑NPs are biocompatible and stable over time ((Figure S11 and S12, Supporting Information), even at 10 µM for 72 h, and that they do not induce IAV infection or alter viral RNA dynamics (Figure S13 and S14, Supporting Information) as determined by cell viability, fluorescence, and qRT‑PCR assays. With these properties validated, we then tested their ratiometric imaging performance. Remarkably, after 6 h co-incubation with VMR-NPs, the FL_ratio_ in IAV-infected A549 cells was higher than that in uninfected controls in a dose-dependent manner, showing a 1.16-fold increase at a multiplicity of infection (MOI) of 10⁻³ and a 2.05-fold increase at an MOI of 10 (Fig. 4a, b). Principal components analysis (PCA) further demonstrated separable clustering among uninfected cells and cells infected at different viral titers, supporting the sensitivity of the ratiometric signal to both infection-associated and dose-dependent changes (Fig. 4c). In addition, correlation analysis revealed a strong linear relationship (R**²** = 0.99) between FL_ratio_ and viral titers. Impressively, VMR-NPs remained responsive even at an MOI of 10⁻⁶, and the FL_ratio_ of infected cells was 1.25-fold higher than that of uninfected cells at 48 hpi (Figure S15, Supporting Information). Additional experiments showed that, although both IAV infection and LPS increased cytosolic APE1 levels, obvious FL_ratio_ changes were observed only in the VMR-NPs + IAV group and were attenuated by leptomycin B (LMB, an inhibitor of the extranuclear migration of APE1) treatment (Figure S16 and S17, Supporting Information). Together, these results indicate that VMR-NPs provide sensitive ratiometric responses for distinguishing infected from uninfected cells and reflecting infection-associated changes, primarily through vRNA-triggered activation with APE1-assisted signal amplification.

We next investigated the time-dependent fluorescence behavior of VMR-NPs under infected conditions, we infected A549 cells with a low viral dose (MOI = 10⁻³) and subjected to serial FL imaging for 72 h. As shown in Fig. 4d, e, the FL_ratio_ gradually increased over time and reached a plateau at approximately 48 h post-infection. To provide orthogonal biological validation, we performed 50% tissue culture infective dose (TCID₅₀) assays on matched samples over the same time course. The resulting viral growth kinetics were broadly consistent with the temporal change in FL_ratio_ (Fig. 4f). Together, these findings support the use of VMR-NPs for fluorescence assessment of infection-associated IAV signals at different time points in host cells.

### In vivo biodistribution and pulmonary retention behavior of VMR-NPs

**Fig. 5:**
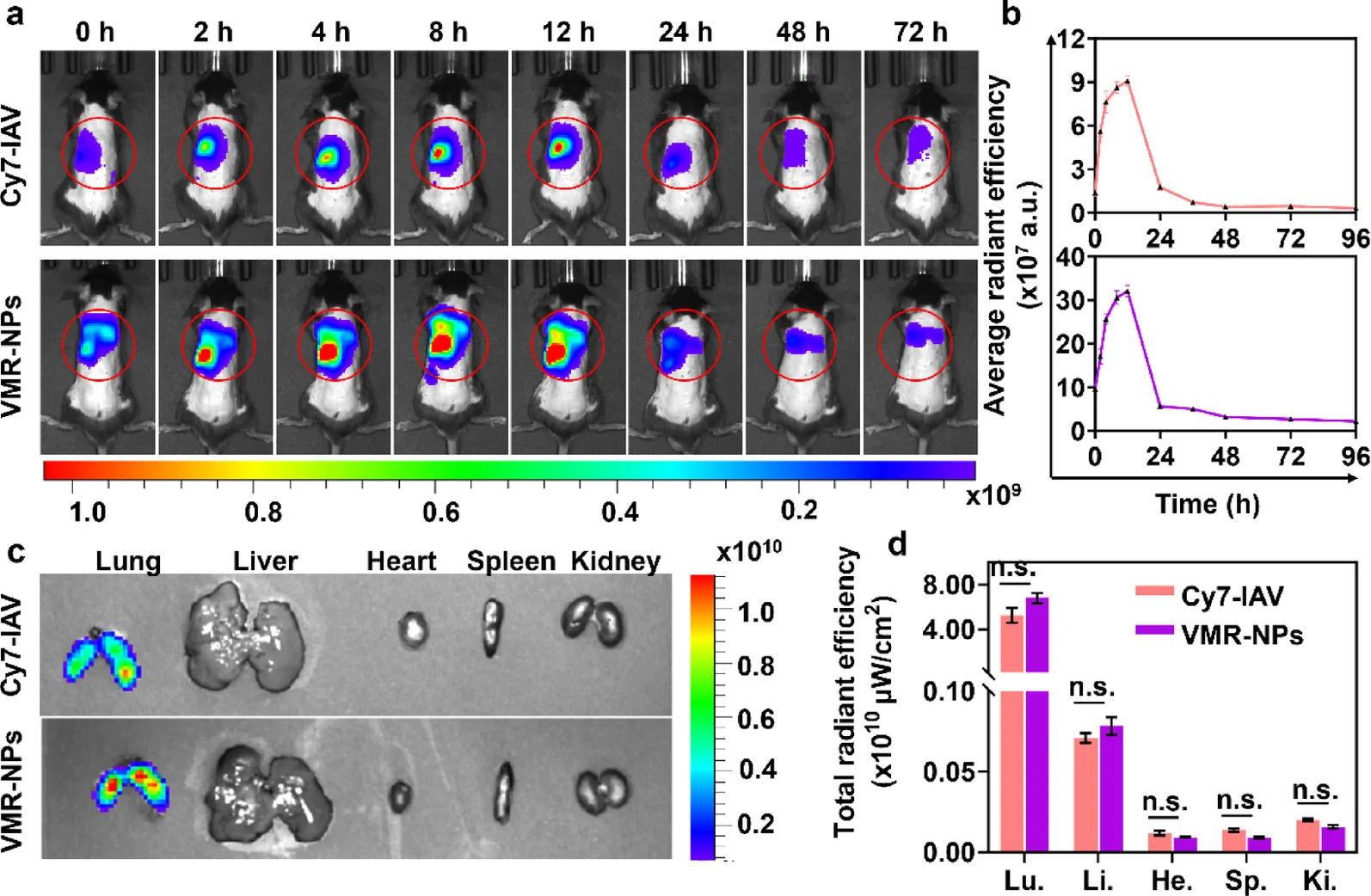
In vivo biodistribution and pulmonary retention kinetics of vmr-nps after intratracheal administration. a) In vivo NIR FL imaging of mice after intratracheal administration of IAVs or VMR-NPs. b) Quantification of average radiant efficiency in the lung region for Cy7-labeled IAVs and VMR-NPs at the indicated time points. c) Ex vivo FL imaging of dissected organs from mice in different treatment groups. d) Total radiant efficiency of IAVs and VMR-NPs in isolated mouse lungs. Data are presented as mean ± standard deviation (n = 3). Statistically significant differences between groups were identified by unpaired two-tailed Student’s t-test. n.s. not significant.

 Encouraged by the in vitro performance of VMR-NPs, we next investigated their in vivo biodistribution and pulmonary retention behavior using the Cy5/Cy7 NIR FRET fluorescent pairs. As a comparator, IAV particles were directly labeled with Cy7 via amidation [5, 26]. Both VMR-NPs and Cy7-labeled IAVs were administered intratracheally to mice, and the resulting lung-associated fluorescence signals were monitored over 72 h. NIR fluorescence signals in the Cy7 channel was acquired using an in vivo small-animal NIR imaging system under 650 nm laser irradiation (0.005 W cm⁻²). As shown in Fig. 5a, b, the two formulations displayed broadly similar lung-associated retention kinetics in mice, characterized by gradual accumulation during the initial 12 hpi, peak FL intensity at 12 hpi, and a subsequent progressive decline consistent with the natural clearance kinetics of respiratory pathogens [27–29]. To further evaluate organ distribution after respiratory delivery, the lungs, liver, heart, spleen, and kidneys were harvested for ex vivo imaging. Strong and comparable FL_Cy7_ signals were detected in the lungs in both groups, whereas signals in other organs were negligible (Fig. 5c–d). It is worth mentioning that HA immunofluorescence staining of lung tissues at 72 h showed persistent and distinct HA signals with a distribution comparable to native IAV (Figure S18, Supporting Information), confirming the stability of the HA coating on VMR-NPs in the pulmonary environment. Collectively, these data demonstrate that VMR-NPs display pulmonary accumulation and retention behavior comparable to those of Cy7-labeled IAV after intratracheal administration, supporting their use as an IAV virosome-assisted sensing platform with IAV-like lung distribution and retention in vivo behavior.

### Ratiometric NIR FL imaging of infection-associated IAV burden In Vivo

**Fig. 6:**
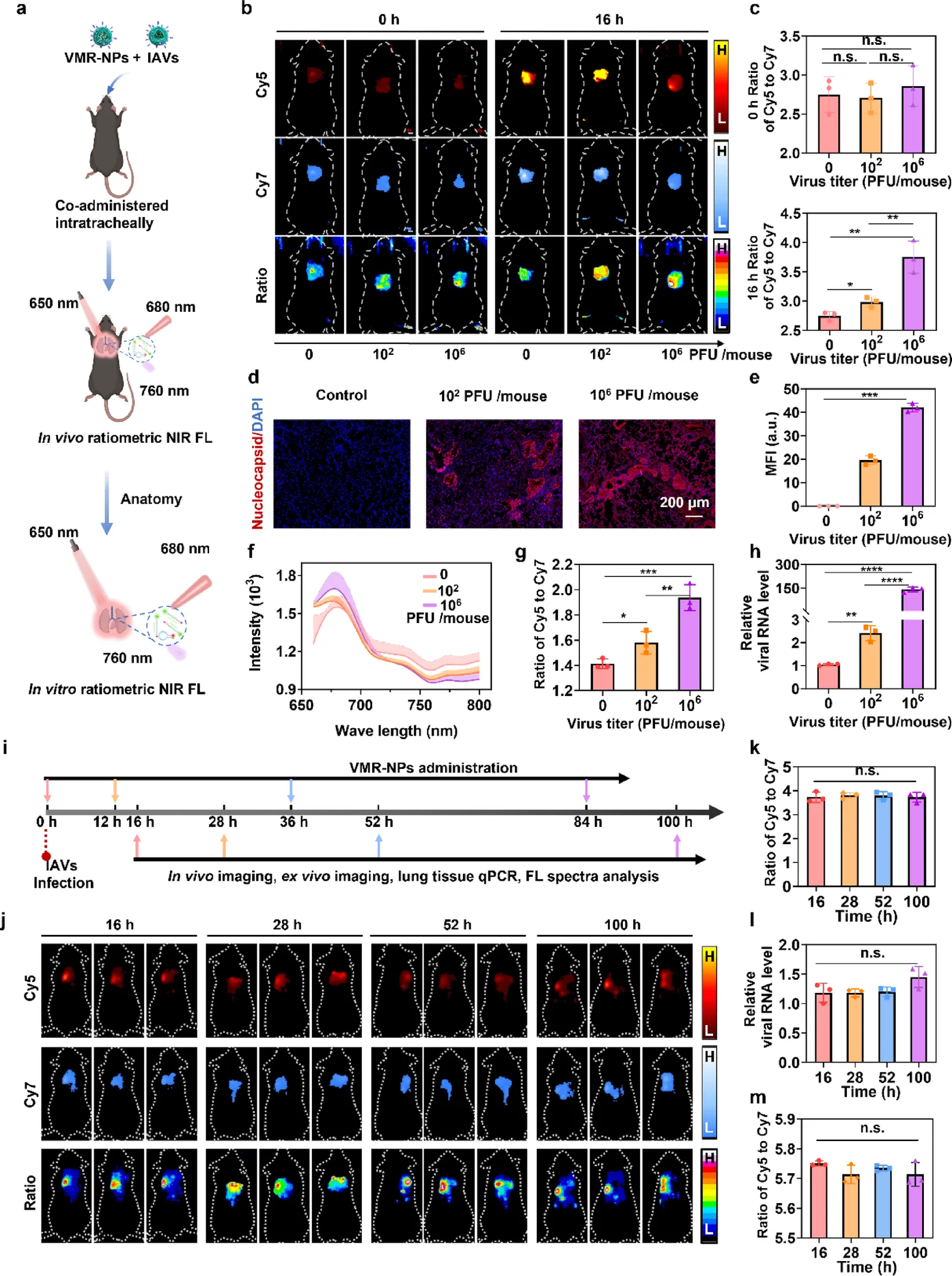
In vivo ratiometric imaging of infection-associated IAV burden using VMR-NPs. **a**) Schematic illustration of the dose-dependent in vivo imaging experiment. **b-c**) In vivo ratiometric FL images (**b**) and quantitative analysis (**c**) of mice infected with different IAV doses, detected by VMR-NPs at 0 and 16 hpi. **d-e**) Representative FL images (**d**) and quantification (**e**) of lung sections from mice infected with various IAV doses at 16 hpi. **f-g**) FL spectra (**f**) and quantitative analysis (**g**) of VMR-NPs in single-cell suspensions isolated from lungs of IAV-infected mice at 16 hpi. **h**) RT-qPCR analysis of viral RNA levels in harvested lungs of IAV-infected mice at 16 hpi. **i**) Timeline of the in vivo serial low-dose infection imaging experiment. **j-k**) In vivo ratiometric FL images (**j**) and quantification (**k**) of VMR-NPs for serial in vivo fluorescence assessment. **l**) RT-qPCR analysis of vRNA levels in IAV-infected mouse lungs, corresponding to the serial in vivo fluorescence measurements in (**j**). **m**) FL_ratio_ quantification of VMR-NPs in single-cell suspensions isolated from lungs of IAV-infected mice. Data are presented as mean ± standard deviation (*n* = 3). Statistically significant differences between groups were identified by one-way ANOVA with Sidak’s multiple comparisons test (**c**,** f-h**, and **k-m**). Date in panel **e**) were identified by unpaired two-tailed Student’s t-test. n.s. not significant, *P < 0.05, **P < 0.01, ***P < 0.001, ****P < 0.0001

To assess the ability of VMR-NPs to report infection-associated IAV burden in vivo, C57BL/6 mice were intratracheally administered varying doses of IAVs along with excess VMR-NPs (10 µM), and the VMR-NP signal was recorded by in vivo small animal NIR bioimaging system (Fig. 6a). NIR FL signals in both Cy5 and Cy7 channels increased significantly at 16 hpi, reflecting pulmonary enrichment of VMR-NPs (this time point includes a 4 h lysosomal escape phase following 12 h of optimal accumulation). Notably, the FL_ratio_ remained essentially unchanged in healthy mice (non-infected controls) (Fig. 6b, c and Figure S19, Supporting Information), indicating high structural stability of VMR-NPs in vivo. In contrast, IAV-infected mice exhibited differential signal dynamics, with Cy5 channel intensity increasing more prominently relative to the Cy7 channel and leading to IAVs dose-dependent elevation of the FL_ratio_. Specifically, the NIR FL_ratio_ of IAV-infected mice were 1.15-fold (10^2^ plaque-forming units per mouse, PFU/mouse) and 1.53-fold (10^6^ PFU/mouse) higher than those in healthy controls, respectively (Fig. 6c). Consistent trends were also observed in ex vivo lung tissue imaging (Figure S20, Supporting Information) and single-cell suspensions prepared from infected lung tissues (Fig. 6f, g). To further validate the in vivo fluorescence readout, the IAV-infected lung tissues were collected for immunofluorescence staining (Fig. 6d, e) and viral RNA in harvested lungs was analyzed by reverse transcription quantitative polymerase chain reaction (RT-qPCR) (Fig. 6h). Both assays showed trends consistent with the in vivo FL_ratio_ signal.

To further investigate the time-dependent in vivo fluorescence behavior of VMR-NPs under low-dose infection conditions, we established a low-dose IAV (10^2^ PFU/mouse) model and performed serial imaging up to 100 h. In this experiment, VMR-NPs were administered 16 h before each imaging time point to ensure maximal pulmonary accumulation and excess probe availability at the time of measurements (Fig. 6i). The results showed no significant changes in FL_Cy5_, FL_Cy7_, or FL_ratio_ over the 96 h observation period (Fig. 6j, k; Figure S21, Supporting Information). Furthermore, consistent trends were also obtained from ex vivo lung imaging (Figure S22, Supporting Information), RT-qPCR analysis (Fig. 6l), and fluorescence analysis of VMR-NPs in excised lung tissues (Fig. 6m and Figure S23, Supporting Information). Notably, a parallel IAV‑alone qRT‑PCR control revealed a similarly stable pulmonary viral RNA profile, arguing against the possibility that the stable signals in the IAV + VMR‑NPs group arose from nanoparticle‑mediated interference. Under this low‑dose condition, the limited viral expansion is likely attributable to local respiratory clearance during the observation window (Fig. S24). Together, these results verified that VMR-NPs enable serial in vivo assessment of infection-associated IAV signals at different time points under low-dose viral conditions.

## Conclusion

In summary, VMR-NPs represent a universal platform for the spatiotemporal visualization of IAV infection in vivo. By synergistically integrating IAV virosome-mediated host-cell recognition, vRNA-triggered signal activation, and APE1-assisted signal amplification, VMR-NPs exhibit IAV-like cellular uptake and intracellular trafficking behavior, while also showing IAV-like pulmonary distribution and retention in vivo after intratracheal administration. On this basis, VMR-NPs enable sensitive ratiometric fluorescence assessment of infection-associated IAV signals across different infection levels and time points in vitro and in vivo. Beyond IAVs, the modular architecture of VMR-NPs provides a versatile blueprint for the development of imaging tools targeting other viral pathogens. These findings support the use of virosome-assisted ratiometric nanoprobes as experimental tools for studying virus–host interaction-related molecular events in biological settings.

## Supplementary Information

Supplementary Material 1

Supplementary Material 2

## Data Availability

All data generated or analyzed during this study are included in the manuscript or supplementary information files. Additional information is available from the corresponding author upon reasonable request.

## Abbreviations

IAVs: Influenza viruses
VMR-NPs: virosome-masked ratiometric nanoprobes
VIROs: Virosomes
FRET: Förster resonance energy transfer
MB: Molecular beacon
vRNA: viral RNA
AP: Apurinic/apyr-imidinic
APE1: Apurinic/apyr-imidinic (AP) endonuclease 1
HA: Hemagglutinin
FL: Fluorescence
IVIS: In vivo imaging system
NA: Neuraminidase
ICQ: Intensity correlation quotient
MSD: Mean-squared displacement
PCA: Principal components analysis
RT-qPCR: Reverse transcription quantitative polymerase chain reaction

## References

[1] Yamaji R, Zhang W, Kamata A, Adlhoch C, Swayne DE, Pereyaslov D, Wang D, Neumann G, Pavade G, Barr IG, et al. Pandemic risk characterisation of zoonotic influenza A viruses using the Tool for Influenza Pandemic Risk Assessment (TIPRA). Lancet Microbe. 2025;6:100973. 10.1016/j.lanmic.2024.100973 PMC11876097 39396528

[2] Torres Montaguth OE, Buddle S, Morfopoulou S, Breuer J. Clinical metagenomics for diagnosis and surveillance of viral pathogens. Nat Rev Microbiol. 2026;24:61–75. 10.1038/s41579-025-01223-5 40804538

[3] Holdener C, Jiang S, Sutherland DM, Griswold KA, Dermody TS, Parker JSL, De Vlaminck I. Spatial Transcriptomics to Study Virus-Host Interactions. Annual Rev Virol. 2025;12:379–99. 10.1146/annurev-virology-092623-104926 PMC12683572 40523107

[4] Ouyang A, Chen T, Feng Y, Zou J, Tu S, Jiang M, Sun H, Zhou H. The Hemagglutinin of Influenza A Virus Induces Ferroptosis to Facilitate Viral Replication. Adv Sci. 2024;11:2404365. 10.1002/advs.202404365 PMC11497066 39159143

[5] Liu S-L, Wang Z-G, Xie H-Y, Liu A-A, Lamb DC, Pang D-W. Single-Virus Tracking: From Imaging Methodologies to Virological Applications. Chem Rev. 2020;120:1936–79. 10.1021/acs.chemrev.9b00692 PMC7075663 31951121

[6] Seah D, Cheng Z, Vendrell M. Fluorescent Probes for Imaging in Humans: Where Are We Now? ACS Nano. 2023;17:19478–90. 10.1021/acsnano.3c03564 PMC10604082 37787658

[7] Broich L, Wullenkord H, Osman MK, Fu Y, Müsken M, Reuther P, Brönstrup M, Sieben C. Single influenza A viruses induce nanoscale cellular reprogramming at the virus-cell interface. Nat Commun. 2025;16:3846. 10.1038/s41467-025-58935-8 PMC12032206 40280912

[8] Yu J, Zhang Y. Activatable Fluorescent Probes for In Vivo and Ex Vivo Dynamic Profiling of Virus-Infected Macrophages. J Am Chem Soc. 2025;147:41079–86. 10.1021/jacs.5c15321 41122937

[9] Cosco ED, Bogyo M. Recent advances in ratiometric fluorescence imaging of enzyme activity in vivo. Curr Opin Chem Biol. 2024;80:102441. 10.1016/j.cbpa.2024.102441 PMC11164639 38457961

[10] Tang Y, Li Y, He C, Wang Z, Huang W, Fan Q, Liu B. NIR-II-excited off-on-off fluorescent nanoprobes for sensitive molecular imaging in vivo. Nat Commun. 2025;16:278. 10.1038/s41467-024-55096-y PMC11696168 39747854

[11] Kelly B, Boudreau JE, Beyea S, Brewer K. Molecular imaging of viral pathogenesis and opportunities for the future. npj Imaging. 2025;3:3. 10.1038/s44303-024-00056-w PMC11761071 39872292

[12] Wang X, Ding Q, Groleau RR, Wu L, Mao Y, Che F, Kotova O, Scanlan EM, Lewis SE, Li P, et al. Fluorescent Probes for Disease Diagnosis. Chem Rev. 2024;124:7106–64. 10.1021/acs.chemrev.3c00776 PMC11177268 38760012

[13] Liu S-L, Zhang Z-L, Tian Z-Q, Zhao H-S, Liu H, Sun E-Z, Xiao GF, Zhang W, Wang H-Z, Pang D-W. Effectively and Efficiently Dissecting the Infection of Influenza Virus by Quantum-Dot-Based Single-Particle Tracking. ACS Nano. 2012;6:141–50. 10.1021/nn2031353 22117089

[14] Xie B, Xu S, Sivasankar S. Outside-in engineering of cadherin endocytosis using a conformation strengthening antibody. Nat Commun. 2025;16:1157. 10.1038/s41467-025-56478-6 PMC11779849 39881179

[15] Sheng C, Zhao J, Di Z, Huang Y, Zhao Y, Li L. Spatially resolved in vivo imaging of inflammation-associated mRNA via enzymatic fluorescence amplification in a molecular beacon. Nat Biomedical Eng. 2022;6:1074–84. 10.1038/s41551-022-00932-z 36050523

[16] Yu C, Chen H-J, Liu H-Y, Ning D, Wang L, Shi X-H, Wang Z-G, Pang D-W, Liu S-L. Dual-lock-and-key virus-mimicking nanoprobes for ultra-high accurate and sensitive imaging of viral infections in vivo. Nano Today. 2024;59:102527.

[17] Amacker M, Smardon C, Mason L, Sorrell J, Jeffery K, Adler M, Bhoelan F, Belova O, Spengler M, Punnamoottil B, et al. New GMP manufacturing processes to obtain thermostable HIV-1 gp41 virosomes under solid forms for various mucosal vaccination routes. npj Vaccines. 2020;5:41. 10.1038/s41541-020-0190-9 PMC7235025 32435515

[18] Stegmann T, Morselt HW, Booy FP, van Breemen JF, Scherphof G, Wilschut J. Functional reconstitution of influenza virus envelopes. EMBO J. 1987;6:2651–9. 10.1002/j.1460-2075.1987.tb02556.x PMC553686 3678202

[19] Asadi K, Gholami A. Virosome-based nanovaccines; a promising bioinspiration and biomimetic approach for preventing viral diseases: A review. Int J Biol Macromol. 2021;182:648–58. 10.1016/j.ijbiomac.2021.04.005 PMC8049750 33862071

[20] Maslanka Figueroa S, Veser A, Abstiens K, Fleischmann D, Beck S, Goepferich A. Influenza A virus mimetic nanoparticles trigger selective cell uptake. Proc Natl Acad Sci. 2019;116:9831–6. 10.1073/pnas.1902563116 PMC6525500 31036631

[21] Du L, Hou Y-N, Fu D-D, Li J, Ao J, Ma A-X, Wan Q-Q, Wang Z-G, Liu S-L, Zhang L-J, Pang D-W. Revealing Different Pathways for Influenza A Virus To Reach Microtubules after Endocytosis by Quantum Dot-Based Single-Virus Tracking. ACS Nano. 2024;18:23090–103. 10.1021/acsnano.4c05261 39143650

[22] Liu S-L, Zhang L-J, Wang Z-G, Zhang Z-L, Wu Q-M, Sun E-Z, Shi Y-B, Pang D-W. Globally Visualizing the Microtubule-Dependent Transport Behaviors of Influenza Virus in Live Cells. Anal Chem. 2014;86:3902–8. 10.1021/ac500640u PMC4004192 24678700

[23] Qin C, Li W, Li Q, Yin W, Zhang X, Zhang Z, Zhang X-E, Cui Z. Real-time dissection of dynamic uncoating of individual influenza viruses. *Proceedings of the National Academy of Sciences* 2019, 116:2577–2582. 10.1073/pnas.1812632116 PMC6377448 30626642

[24] Benton DJ, Gamblin SJ, Rosenthal PB, Skehel JJ. Structural transitions in influenza haemagglutinin at membrane fusion pH. Nature. 2020;583:150–3. 10.1038/s41586-020-2333-6 PMC7116728 32461688

[25] Bonazza S, Courtney DG. Influenza A virus RNA localisation and the interceding trafficking pathways of the host cell. PLoS Pathog. 2025;21:e1013090. 10.1371/journal.ppat.1013090 PMC12017568 40267083

[26] Ozcelik D, Jain A, Stambaugh A, Stott MA, Parks JW, Hawkins A, Schmidt H. Scalable Spatial-Spectral Multiplexing of Single-Virus Detection Using Multimode Interference Waveguides. Sci Rep. 2017;7:12199. 10.1038/s41598-017-12487-0 PMC5610187 28939852

[27] Pan W, Dong Z, Li F, Meng W, Feng L, Niu X, Li C, Luo Q, Li Z, Sun C, Chen L. Visualizing influenza virus infection in living mice. Nat Commun. 2013;4:2369. 10.1038/ncomms3369 PMC3778511 24022374

[28] Ueki H, Wang IH, Fukuyama S, Katsura H, da Silva Lopes TJ, Neumann G, Kawaoka Y. In vivo imaging of the pathophysiological changes and neutrophil dynamics in influenza virus-infected mouse lungs. *Proceedings of the National Academy of Sciences* 2018, 115:E6622-E6629. 10.1073/pnas.1806265115 PMC6048509 29941581

[29] Ullah I, Beaudoin-Bussières G, Symmes K, Cloutier M, Ducas E, Tauzin A, Laumaea A, Grunst MW, Dionne K, Richard J, et al. The Fc-effector function of COVID-19 convalescent plasma contributes to SARS-CoV-2 treatment efficacy in mice. Cell Rep Med. 2023;4:100893. 10.1016/j.xcrm.2022.100893 PMC9799175 36584683

