# Supplementary material for: Virosome-masked ratiometric nanoprobes for in vivo dynamic imaging of influenza a virus infection
Source: J Nanobiotechnology. 2026 Jul 21;24:963. doi: 10.1186/s12951-026-04819-y (PMC13673603; doi:10.1186/s12951-026-04819-y)
Supplement: Supplementary file 1 [file 12951_2026_4819_MOESM2_ESM.docx]

**Supplementary Information**

**Virosome-Masked Ratiometric Nanoprobes for In Vivo Dynamic Imaging of Influenza A Virus Infection**

Ruiqi Ming^1^, Yue Wang^2^, Zewei Yan^3^, Shujun Liu^1^, Zhongjie Wang^1^, Hanlin Chen^1^, Yingjie Shi^2^, Yuantian Jing^1^, Rui Zhang^1^, Shasha Peng^1^, Jiajie Tian^1^, Yimei Pan^1^, Wenfeng Xu^2^, Pengfei Jin^2^*, Li-Li Huang^1^*

^1^School of Medical Science and Engineering, Beijing Institute of Technology, Beijing 100081, P. R. China

^2^Department of Pharmacy, Beijing Hospital, National Center of Gerontology; Institute of Geriatric Medicine, Chinese Academy of Medical Sciences; Beijing Key Laboratory of Assessment of Clinical Drugs Risk and Individual Application, Beijing 100730, P. R. China

^3^School of Life Sciences, Inner Mongolia Normal University, Hohhot 010022, P. R. China

E-mail: Li-Li Huang, [llhuang@bit.edu.cn](mailto:llhuang@bit.edu.cn); Pengfei Jin, [j790101@163.com](mailto:j790101@163.com)

**Content:**

**Figs. S1 to S15**

**Movies. S1 to S3**

**Figure S1.** Normalized fluorescence spectra of R-NPs with excitation at 520 nm. (a) R-NPs alone (1 μM); (b) R-NPs (1 μM) and complementary vRNA (2 μM).

**Figure S2.** Sequence-specific response of R-NPs toward target vRNA. a) Fluorescence spectra of R-NPs and non-targeting R-NPs (nR-NPs) (0.5 μM) in the absence or presence of target vRNA (0.15 μM). b) Quantitative analysis of the Cy3/Cy5 fluorescence ratio corresponding to panel (a). Data are presented as mean ± SD (n = 3). Statistical significance was analyzed by one-way ANOVA with Sidak’s multiple comparisons test. n.s., not significant; ****P < 0.0001.

**Figure S3.** Effect of APE1 concentration on signal amplification. Fluorescence spectra of R-NPs (0.5 μM) responding to IAV vRNA (0.05 μM) with different APE1 concentrations.

**Figure S4.** APE1-assisted detection sensitivity of R-NPs toward target vRNA in solution. (a) Fluorescence spectra of R-NPs (0.5 μM) responding to different concentrations of IAV vRNA concentration (0-400 nM) with APE1 (1.4μg/mL). (b) Linear relationship between the Cy3/Cy5 fluorescence ratio and vRNA concentration over the full range of 0–400 nM. (c) The linear calibration curves at low vRNA concentration (0-50 nM) for the LOD determination. Mean ± standard deviations, n=3.

**Figure S5.** Level of genetic material in VMR-NPs, VIROs and IAVs.

**Figure S6**. Western blot analysis of HA and NA proteins in VMR-NPs, VIROs and IAVs.

**Figure S7.** Loading efficiency (a) and encapsulation efficiency (b) of R-NPs in VMR-NPs. Data are presented as mean ± standard deviation (n = 3).

**Figure S8.** The stability of VIROs during 7 days in 10% FBS (v/v). Data are presented as mean ± standard deviation (n = 3).

**Figure S9.** Particle concentration stability of VIROs during 7 days in 10% FBS (v/v). Data are presented as mean ± standard deviation (n = 3).

**Figure S10.** Snapshot of IAVs, VMR-NPs or LR-NPs propagation in live cells at different time points. Scale bars, 5 µm.

**Figure S11.** Evaluation of the biocompatibility of VMR-NPs in A549 cells using the CCK-8 assay. Data are presented as mean ± standard deviation (n = 4). Statistically significant differences between groups were identified by one-way ANOVA with Sidak’s multiple comparisons test. n.s. not significant.

**Figure S12.** Stability analysis of VMR-NPs in cells. a) Fluorescence images of cells infected without virus at different time points were detected by the VMR-NPs. scale bar: 200 μm. b) Quantitative analysis of the results in panel (a).

**Figure S13.** Relative intracellular viral RNA levels in cells treated with VMR-NPs or blank control at different time points post-infection. Data are presented as mean ± SD (n = 3). Statistically significant differences between groups were identified by one-way ANOVA with Sidak’s multiple comparisons test. n.s. not significant.

**Figure S14.** Time-course RT-qPCR analysis of viral RNA levels in A549 cells infected with IAV alone or treated with IAV together with VMR-NPs for 6, 12, 24, and 48 h. Data are presented as mean ± SD (n = 3). Statistically significant differences between groups were identified by one-way ANOVA with Sidak’s multiple comparisons test. n.s. not significant.

**Figure S15.** In vitro detection of IAV concentration. a) Fluorescence images of A549 cells infected with different titers of virus detected by VMR-NPs at 48 hpi. scale bar: 200 μm. b-c) Quantitative analysis of the results in panel (a). Data are presented as mean ± standard deviation (n = 3).

**Figure S16.** Cytosolic APE1 levels in A549 cells. a) Representative western blot images of cytosolic APE1 in A549 cells under different treatments for 6 h. b) Quantification of relative cytosolic APE1 expression shown in panel (a). Data are presented as mean ± standard deviation (n = 3). Statistically significant differences between groups were identified by one-way ANOVA with Sidak’s multiple comparisons test. n.s. not significant, *P < 0.05, ****P < 0.0001.

**Figure S17.** Fluorescence responses of VMR-NPs in A549 cells. a) Representative merged confocal fluorescence images of A549 cells under different treatments for 6 h. Scale bar: 200 μm. b) Corresponding single-cell fluorescence ratio analysis for the groups shown in panel (a). Data are presented as mean ± standard deviation (n = 3). Statistically significant differences between groups were identified by one-way ANOVA with Sidak’s multiple comparisons test. n.s. not significant, ****P < 0.0001.

**Figure S18.** HA immunofluorescence staining of lung tissues 72 h after intratracheal administration of VMR-NPs or IAV. a) Representative immunofluorescence images of HA staining in lung sections collected 72 h after intratracheal administration of VMR-NPs or IAV. Distinct HA fluorescence signals remained detectable in both groups. b) Quantification of HA fluorescence intensity in lung sections shown in panel (a). Data are presented as mean ± standard deviation (n = 3). Statistically significant differences between groups were identified by one-way ANOVA with Sidak’s multiple comparisons test. n.s. not significant.

**Figure S19.** Quantitative analysis of fluorescence signals in Cy5 and Cy7 channels of VMR-NP in vivo at 0 h (a-b) and 16 h (c-d) post intratracheal administration. Data are presented as mean ± standard deviation (n = 3). Statistically significant differences between groups were identified by one-way ANOVA with Sidak’s multiple comparisons test. n.s. not significant, *P < 0.05, **P < 0.01.

**Figure S20.** Ex vivo fluorescence images (a) and quantitative FL_ratio_ analysis (b) of VMR-NPs in lungs excised from mice treated with different IAV titers. Data are presented as mean ± standard deviation (n = 3). Statistically significant differences between groups were identified by one-way ANOVA with Sidak’s multiple comparisons test. n.s. not significant, *P < 0.05, ***P < 0.001.

**Figure S21.** Quantitative analysis of fluorescence signals in Cy5 and Cy7 channels of VMR-NPs at different time points. Data are presented as mean ± standard deviation (n = 3). Statistically significant differences between groups were identified by one-way ANOVA with Sidak’s multiple comparisons test. n.s.: not significant.

**Figure S22.** Ex vivo fluorescence images of lungs excised from mice after different time points. Data are presented as mean ± standard deviation (n = 3). Statistically significant differences between groups were identified by one-way ANOVA with Sidak’s multiple comparisons test. n.s. not significant.

**Figure S23.** In vitro FL detection of IAV load in mouse lungs at different time points.

**Figure S24.** Time-course RT-qPCR analysis of viral RNA levels in mouse lungs infected with IAV alone for 16, 28, 52, and 100 h. Data are presented as mean ± SD (n = 3). Statistically significant differences between groups were identified by one-way ANOVA with Sidak’s multiple comparisons test. n.s. not significant.

**Movies. S1 to S3.** Real-time tracking of movement behaviors of DiI-IAVs (Movie. S1), VMR-NPs (Movie. S2) and LR-NPs (Movie. S3) in A549 cells.
